# Follow-Up of a Rare Case of Eosinophilic Gastroenteritis Associated with Persistent Blood Eosinophilia and Multiple Food Allergies

**DOI:** 10.3390/diagnostics12061381

**Published:** 2022-06-02

**Authors:** Polliana Mihaela Leru, Vlad Florin Anton, Ioana Adriana Muntean, Carmen Daniela Neagoe, Dumitru Matei

**Affiliations:** 1 Clinical Department 5, Carol Davila University of Medicine and Pharmacy, 050474 Bucharest, Romania; dumitru.matei@umfcd.ro; 2 Department of Allergology and Immunology, Colentina Clinical Hospital, 020125 Bucharest, Romania; antonvladflorin@gmail.com; 3 Department of Allergology and Immunology, Iuliu Hatieganu University of Medicine and Pharmacy, 400347 Cluj Napoca, Romania; adriana.muntean@umfcluj.ro; 4 Department of Internal Medicine, University of Medicine and Pharmacy, 200349 Craiova, Romania; dananeagoe2014@gmail.com

**Keywords:** eosinophilic gastrointestinal disorders, eosinophilic gastroenteritis, blood eosinophilia, food allergies, elimination diet

## Abstract

Eosinophilic gastroenteritis (EGE) is a subgroup of the eosinophilic gastro-intestinal disorders (EGIDs), characterized by eosinophilic infiltration and chronic inflammation of the gastrointestinal tract. These are rare diseases with still incompletely elucidated causes and mechanisms, with frequently delayed diagnosis and variable outcome. Despite increased interest in eosinophilic diseases in recent years, fewer data have been published on EGE and no standardized diagnostic and therapeutic approach exists. This paper reports the case of a young male patient diagnosed with EGE in 2017 based on clinical and histopathological criteria and constantly monitored during five years. Besides gastrointestinal eosinophilic infiltration, biopsies also revealed eosinophilic infiltration of the oesophagus, despite no declared characteristic oesophageal symptoms. We found increased specific IgE to multiple foods and progressive blood hypereosinophilia which preceded EGE diagnosis by three years. The EGE management included selective dietary restrictions and pharmacologic therapy based on daily budesonide non-enteric coated tablets, proton pumps inhibitors, antihistamines, cromoglycate, correction of iron, calcium and vitamin D deficiencies. The clinical outcome was good, while blood eosinophilia and endoscopic appearance remained almost unchanged. After one year the patient complained of respiratory symptoms suggesting asthma, needing continuous combined inhaled therapy. The reported case is illustrative for complex presentation, diagnosis and outcome of a rare case of mucosal chronic EGE associated with oesophageal involvement, peripheral eosinophilia, multiple food allergies and asthma.

## 1. Introduction

Eosinophilic gastrointestinal disorders (EGIDs) represent a group of rare diseases characterized by eosinophilic infiltration and chronic inflammation of the gastrointestinal tract [1]. Eosinophilic gastroenteritis (EGE) is due to the eosinophilic infiltration that involves single or multiple segments from the stomach to the colon, with or without oesophageal involvement [2]. EGE is distinct from eosinophilic esophagitis (EoE) which is characterized by eosinophilic infiltration of the oesophagus. An increasing interest in EGIDs was noted in recent decades, with eosinophilic esophagitis (EoE) being the most prevalent and better characterized. The diagnosis of EGE is frequently delayed, mostly due to variable clinical picture, inconsistent correlation with histopathologic examination and the association of blood eosinophilia which needs exclusion of many other diseases [3]. Eosinophilic gastroenteritis (EGE) is considered a rare and heterogeneous disease, with a still unclear pathophysiologic mechanism beyond the eosinophilic involvement and no standardized diagnostic and therapeutic approach exists [4].

EGE was first described by Kaijser in 1937 and about 300 cases have been reported in the literature so far [5]. The reported prevalence of EGE is about 5.1–5.3/100.000 and seems to be rising in recent years, with female and Caucasian race predilection [6,7]. The disease can occur at any age, from infancy to the seventh decade, with the peak of incidence between the third and the fifth decade of life [8].

The eosinophilic inflammation of the gastrointestinal tract is the result of a mixed IgE-mediated and Th2-dependent immune dysregulation, leading to increased eosinophil recruitment, activation and survival, coupled with antigen specific IgE production [9]. The Th2 associated immune responses are responsible for overproduction of the cytokines IL-4, IL-5, IL-13 and eotaxin, with IL-5 having a major role in eosinophils development and activation [10]. Patients with EGE have frequently co-existing allergic diseases, such as asthma, rhinitis, food and drug allergies, urticaria and eczema, as reported in the study by Mansoor et al. [6]. Atopy is found in about 80% of the patients with EGIDs, including EGE, and more than 50% of cases have blood eosinophilia [11].

There are no gold-standard diagnostic criteria for non-EoE eosinophilic gastrointestinal diseases and the currently accepted approach is based on three types of criteria, suggested by Talley in 1990: gastrointestinal symptoms, endoscopic biopsies showing eosinophilic infiltration of the stomach and small intestine and extensive exclusion of other differential diagnoses of peripheral and/or tissue eosinophilia [2].

The aim of this manuscript is to present the five-year follow-up and outcome of a rare case of eosinophilic gastroenteritis with oesophageal involvement, associated with persistent blood eosinophilia, asthma and multiple food allergies in a young male adult and to outline some relevant diagnostic and problematic aspects reviewed in the literature on this topic. The summary of the data from the initial presentation of the patient were presented in a Letter to the Editor published in 2018 [12].

## 2. Case Report

We report the case of a young man born in Romania in 1985, a present resident of Ireland, who was diagnosed with EGE at the Allergy Department of Colentina Clinical Hospital from Bucharest in October 2017 at age of 32 years and has been constantly monitored since then. He initially presented to our clinic for allergist evaluation due to significant blood hypereosinophilia, persistent gastrointestinal symptoms—nausea, vomiting, diarrhea, flatulence and significant weight loss, with progressive onset about one year before presentation. The patient noticed moderate blood eosinophilia on his regular check-up since 2014, while having no symptoms, but no other laboratory tests were performed due to good health status. He had no personal history of allergies, but his mother has asthma. About one year before presentation, he noticed symptoms aggravation after ingestion of some foods, mainly milk, eggs and wheat. Previous medical evaluation performed by the ambulatory gastroenterologist six months before presentation revealed active gastritis and esophagitis with florid eosinophilic infiltration and carpet of antral polyps, but no specific treatment was given since blood eosinophilia was considered possibly reactive to parasitic infections. Laboratory tests at hospital admission confirmed high blood eosinophilia 5310/μL (49.5% of the total white blood cell). Complementary laboratory tests showed: moderate high serum total IgE, decreased total serum proteins, slight decrease of iron and total serum calcium and insufficient level of 25-hydroxycholecalciferol. The serum level of eosinophil cationic protein (ECP), an important marker of activated eosinophils, was very high (>200 μg/L). We found significant high specific IgE to almost all essential foods: cow milk, wheat, eggs, various meat and seafood (using Immuno-CAP Food Allergy Assay) (see Table 1).

**Figure 1:**
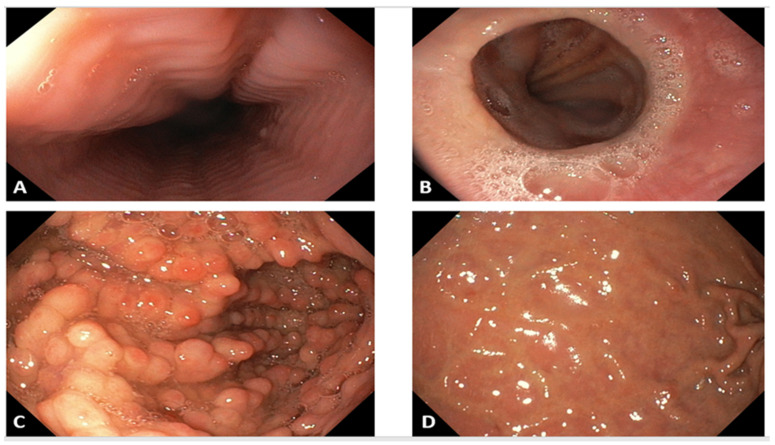
Endoscopic findings of the oesophagus and stomach at EGE diagnosis 2017 (performed at Colentina Clinical Hospital, Gastroenterology Department). (**A**,**B**)—Oesophagus: oesophageal rings (“trachealization” or “crepe paper” aspect) and Schatzky’s ring; (**C**,**D**)—Stomach: carpet of antral polyps >100, covering the entire antrum of various size, with intensely hyperemic friable mucosa. Nodularity and patchy erythema of the gastric body.

**Figure 2:**
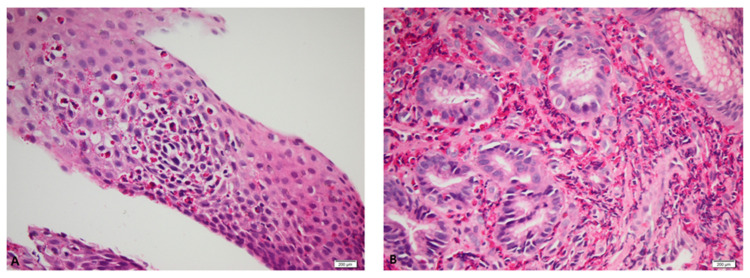
Histopathologic aspects of the oesophageal and gastric mucosa (Pathology Laboratory from Colentina Clinical Hospital). (**A**) Oesophageal mucosa with erosive areas, presenting acanthosis, areas of spongiosis and marked intraepithelial eosinophilic exocytosis with intraepithelial eosinophilic abscesses (96 Eos/HPF); (**B**) Multiple fragments of gastric antral mucosa showed florid infiltration by eosinophils (>200 Eos/HPF); the eosinophils are predominantly located within the lamina propria, with focal areas of involvement of the surface epithelium; there are florid reactive epithelial changes, with focal surface erosion and frequent glandular dilatations; no intestinal metaplasia, dysplasia or malignancy present.

The repeated in vitro allergy diagnosis using the ELISA multiplex method after two years described food and respiratory allergen components and are shown in the Table 2. The endoscopic examination of the upper gastro-intestinal tract with multiple biopsies was repeated in the hospital gastroenterology laboratory and showed notable pathologic changes of the stomach and oesophagus as follows: stomach with carpet of antral polyps >100, of various size, covering the entire antrum, with intensely hyperemic friable mucosa, nodularity and patchy erythema of the gastric body and oesophageal mucosa with “crepe paper” aspect and Schatzki ring (see Figure 1). Multiple biopsies were performed and showed important mucosal eosinophilic infiltration, notably in the gastric mucosa (>200 eosinophils/HPF) and also in the oesophagus (with 96 eosinophils/HPF) (see Figure 2). The aspect of the duodenum was normal and biopsies showed preserved villous architecture, minimal regenerative epithelial changes and moderate inflammatory infiltrate including zonal eosinophilic frequencies (27 Eos/HPF) diffused in the chorion. The endoscopic examination of the small intestine and the colon was performed in another hospital from Ireland and the patient sent us the written report which mentioned normal aspect; no endoscopic images from those segments are available.

In order to exclude other causes of blood and gastrointestinal eosinophilia, we performed stool and blood tests for parasitic infections, serum tryptase, autoantibodies for autoimmune diseases and tumoral markers for neoplasia, which were all negative. The repeated tests for celiac disease were negative and the full body CT scan did not show any significant abnormalities. The primary hypereosinophilic syndrome (HES) was excluded based on the absence of other organ involvement plus bone marrow examination, which showed reactive eosinophilia, with no blasts or atypic cells. We also determined the genetic mutation platelet-derived growth factor receptor alpha—PDGFRA/fibroblast growth factor receptor 1-FGFR1 (F/P), which was negative. The initial established diagnosis was mucosal variant of eosinophilic gastroenteritis and eosinophilic esophagitis associated with blood eosinophilia and multiple food allergies based on the patient’s history. We could not confirm all food allergies using the double-blind oral provocation test (DBOPT) which is the gold-standard in food allergy diagnosis, since no specialized center was available and the patient did not agree.

**Figure 3:**
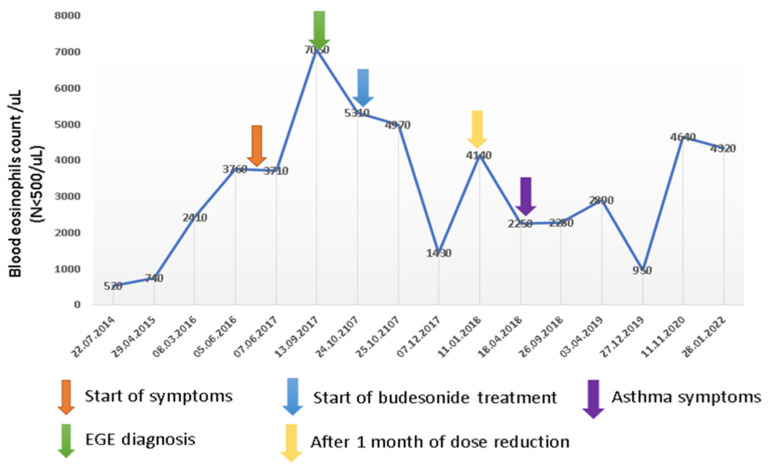
Blood eosinophilia level in 2014–2022.

Considering experts’ recommendations and based on declared relevant food allergies, we first recommended elimination diet, notably avoidance of wheat, cow milk, eggs, peanuts, soy and fish, but this was difficult to keep and results were not satisfactory. Therefore, we initiated corticosteroid treatment with budesonide capsules 9 mg daily, plus, proton pump inhibitors, antihistamines, sodium cromoglycate, calcium and vitamin D3. The clinical outcome after two months was good, with a significant decrease of gastrointestinal symptoms and improvement of health status. Hematologic control showed significant decrease of blood eosinophilia to 1430/μL, correction of serum protein, calcium, iron and 25-OH vitamin D, but the serum level of eosinophil cationic protein (ECP) remained almost unchanged. The patient decided himself to reduce the budesonide dose to 6 mg daily and also tried reintroduction of some foods, thus leading to an increase of blood eosinophilia to 4170/μL. We recommended an increase of budesonide dose to 9 mg daily, a more strict diet with avoidance of allergenic foods and follow-up based on regular hematologic laboratory control, clinical monitoring and endoscopic evaluation after six months. The values of blood eosinophilia up to the most recent control in 2022 are shown in Figure 3.

Endoscopic control was performed in August 2020 and showed almost unchanged aspect of the gastric antral polyps, despite good clinical status and persistence of just mild gastrointestinal symptoms (mostly nausea).

After one year, the patient began to complain of persistent cough, paroxysmal and exercise dyspnoea, nasal symptoms and sinus discharge, notably influenced by the humid and cold climate of Ireland. The spirometry confirmed mild bronchial obstruction, allowing the diagnosis of asthma and persistent rhino-sinusitis. Inhaled treatment with combined budesonide plus formoterol twice daily was initiated and maintained during three years, with good clinical response. The patient continued to check blood tests twice a year and send us the results, as well as the current medical outcome, via online consultations. During the COVID-19 pandemic, in the years 2020 and 2021, we asked about possible complications or unexpected events as well as vaccination status. He safely received a full vaccination scheme with Pfizer BioNTech. The last medical evaluation was performed online in March 2022, when the patient declared mild gastrointestinal symptoms, but bothersome nasal and asthma symptoms, therefore doubling the dose of inhaled therapy was recommended.

## 3. Discussion

This paper describes clinical and histopathological characteristics of an eosinophilic gastroenteritis case followed over five years, which outline the problematic aspects of this rare disease in clinical practice, and also refers to some relevant aspects from the literature on this topic. Since EGE diagnostic criteria and the relationships among the diverse EGIDs are still not clearly defined, there is a need for more case report data regarding the heterogeneous clinical presentation, histopathological aspects, as well as the therapeutic response. We focused on the delay up to the diagnosis confirmation, on the clinical and histopathological pattern, the relevance of blood eosinophilia and the associated allergies, the outcome, the therapeutic response and the prognosis.

EGE diagnosis requires a high index of suspicion. EGE is generally suggested by persistent gastrointestinal symptoms, which are less responsive to usual therapy and frequently associated with blood eosinophilia. Diagnosis requires the exclusion of many other possible causes of a similar clinical picture [13].

The clinical presentation of EGE depends on the affected gastrointestinal segment and the layer depth of the eosinophilic infiltration. According to the classification of Klein from 1970, there are three variants of EGE: mucosal, muscular and serosal, with muscular involvement being the most common [8]. The mucosal EGE type is found in 57–100% of cases and usually presents with abdominal pain, nausea, vomiting, dyspepsia, diarrhea and malabsorption, which may cause anemia, hypoalbuminemia and weight loss [14]. Muscular type is seen in about 30% of cases and may cause bowel wall thickening, intestinal obstruction and bleeding [15]. Serosal EGE is less common; it is estimated to account for up to 9% of EGE cases in Japan and 13% in USA, possibly causing peritoneal irritation, eosinophilic ascites, perforation and is usually accompanied by abundant peripheral eosinophilia [16]. These gastrointestinal symptoms are not specific and can be found in many other organic or functional disorders, such as irritable bowel syndrome, inflammatory intestinal disease, celiac disease or food intolerances. The diagnosis confirmation is done based on endoscopic findings and on histopathologic criteria. The endoscopic normal appearance can be frequently found and the patchy nature of the eosinophilic infiltration imposes multiple random biopsies, a minimum of 5–6, according to most of the authors [17]. The histopathologic criteria of the eosinophilic infiltration consider the cut-off for diagnosis of mucosal EGE at more than 10 eosinophils/HPF for children and more than 20 eosinophils/HPF for adults [18]. Recent data added the following histologic features to support EGE diagnosis: eosinophils greater than 30/HPF for stomach and duodenum, eosinophils in clusters, eosinophils in lamina propria, glandular destruction and intraepithelial eosinophils, eosinophilic cryptitis and crypt abscess [19].

Oesophageal eosinophilic infiltration, even without typical oesophageal symptoms such as dysphagia or food impactation, can be found in EGE, in agreement with evidence from previous studies showing that EGE is typically part of a more generalized EGID and rarely exists in isolation, which has clinical implications for monitoring and treating these patients [9].

Interesting data from the literature suggest a need to consider environmental factors when evaluating tissue eosinophilia, showing that higher counts were found during the peak allergy season and among populations living in some geographical regions with increased allergenic exposure [20].

Blood eosinophilia is present in more than 70% of cases of eosinophilic gastrointestinal diseases and patients with the mucosal predominant type have higher blood eosinophilia levels and greater risk of relapses [21]. High blood eosinophilia at diagnosis is considered an independent predictor of relapses [22]. The absolute eosinophil count (AEC) allows three categories of disease severity: mild with AEC below 1500 eos/μL, moderate with AEC = 1500 − 5000 eos/μL and severe with AEC more than 5000 eos/μL [23].

Allergic diseases are usually accompanied by blood eosinophilia, usually in moderate range and this can be asymptomatic for variable periods of time. The preclinical phase of eosinophilia may suggest the period of hypersensitization to food allergens, before clinically manifested food allergies, with severe forms in rare cases, such as EGE [12]. In this context, blood eosinophilia may not be considered a trivial laboratory parameter and early evaluation and identification of causal disease could improve medium and long-term prognosis. Even though blood eosinophilia does not correlate with symptoms of eosinophilic organic disorder, in some cases, such as the reported one, the clinical diagnosis of EGE was delayed by almost one-year after the onset of gastrointestinal and general symptoms, due to many investigations for blood hypereosinophilia. Total serum IgE is increased in more than two thirds of EGE cases, mostly in atopic patients [24].

The natural history of EGE is not well documented and prognosis depends on risk factors, associated allergic diseases, patient compliance and triggers avoidance [21]. Three long-term progression patterns were described, with persistent disease predominantly observed in the mucosal type, relapsing—remitting disease in the muscular type and non-relapsing disease in the serosal type [25].

Differential diagnosis of EGE has to be done with other diseases manifested with blood hypereosinophilia and associated with eosinophilic infiltration of the gastrointestinal tract and persistent gastrointestinal symptoms. The parasitic infections to be excluded are: Ascaris, Anisakis, Ancylostoma, Enterobius, Strongyloides, Toxocara, Capillaria and Trichinella [16]. Celiac disease is due to intolerance to gliadin, a gluten protein from wheat and is characterized by predominant lymphocyte and not eosinophilic infiltration of the small bowel [26]. Other eosinophilic diseases that need to be considered include: gastric cancer, lymphomas, protein-losing enteropathy, mastocytosis, vasculitis phase of Churg-Strauss syndrome and HES. This last clinical entity has no allergic pattern and is rarely accompanied by gastrointestinal involvement, but other organs, such as lungs and cardio-vascular system can be affected [23].

Possible complications due to EGE are: malabsorption, severe anemia, osteoporosis, gastric or intestinal obstruction and perforation, pyloric stenosis, pancreatitis and severe allergies, such as anaphylaxis. Quality of life can be significantly affected in patients with EGE, mostly in those with chronic symptoms, who face increased physical and psychological stress, fear of complications, financial burden and impaired social and professional life [27]. In the reported case, we have found mild deficits of iron, vitamin D and serum protein, which have been rapidly corrected with therapy, and the patient’s quality of life is moderately affected, due to chronic symptoms and dietary restrictions.

Association with allergic diseases, notably food allergies, is frequently described in EGE patients. Food allergies represent an increasing pathology worldwide in both children and adults, affecting up to 10% of the population and the most allergenic foods are cow’s milk, hen’s egg, wheat, peanuts, tree nuts and seafood [28]. Food allergies represent an important risk factor and trigger for EGE, since food allergens can cross the intestinal mucosa and induce allergic inflammation, consisting of degranulation of mast cells and eosinophils [29]. The precise role of food hypersensitivity is not defined, therefore, many authors consider, similar to EoE, that there is not enough evidence to support routine food allergy testing of patients with EGE for use in a clinical decision [30,31].

The therapeutic approach to eosinophilic disorders of gastrointestinal tract depends on symptoms severity and is based on experts’ recommendations. Dietary restrictions appear to be effective conservative treatment, mainly for the mucosal type of EGE. There are three types of recommended diet, with the six-food elimination diet (6-FED) being the most common, referring to cow’s milk, wheat, hen’s egg, soy, seafood, nuts and peanuts. The seven-food elimination diet (7-FED) also excludes red meat. The second dietary approach is based on the elimination of foods with positive skin or serum tests and the third and the most restrictive is the elemental or amino acid-based diet, which eliminates all potential food allergens [32]. In the reported case, the patient has totally eliminated four of the foods with positive blood tests—wheat, eggs, milk and peanuts, as well as soy and fish, but tolerates pork and chicken meat, despite positive blood tests.

Corticosteroids are the mainstay of pharmacological therapy in patients with moderate to severe disease, due to their proven and prompt effect on eosinophilic inflammation. Budesonide is a locally acting corticosteroid with a high first-pass effect, which minimizes the systemic side effects and confers a better safety profile compared to prednisone [33]. Duration of corticosteroid therapy is not clearly defined. It depends on clinical outcome and relapses often need long-term treatment. In the reported case, the therapeutic response to budesonide was good, but the dose could not be reduced due to the chronic and relapsing pattern of the disease, indicating a probable need for long-term therapy. Various steroid-sparing drugs have been proposed in the literature, such as sodium cromoglycate—a stabilizer of mast cell membranes, antihistamines and montelukast—a selective leukotrienes antagonist [34]. The new and most advanced therapies still under study in EGE include monoclonal antibodies targeting IL-5 such as reslizumab and mepolizumab, anti-IL-5 receptor—benralizumab or anti-IL-4 and IL-13-dupilumab, which have promising results in recent studies [35,36]. Based on the data from the literature showing that IL-13- driving Th2 immunity is locally operational in patients with EGE, treating these patients with dupilumab which is targeting IL-13 and is already approved for treating eosinophilic asthma looks to be a good therapeutic option [9].

## 4. Conclusions

We reported the follow-up during five years of a rare case of eosinophilic gastroenteritis, with histopathological mucosal form, chronic and relapsing outcome, associated with persistent blood eosinophilia, multiple food allergies and asthma. The therapeutic approach was based on selective elimination diet and daily oral budesonide, with constant clinical, laboratory and endoscopic monitoring. We found that despite remission of gastrointestinal symptoms and clear clinical improvement, the endoscopic aspects and blood eosinophilia were slightly influenced, indicating maintenance of the inflammatory process and further possible complications.

The reported case is illustrative for outcome and difficulties in diagnosis, evaluation and long-term management of eosinophilic gastrointestinal disorders. It also underlines the importance of correlation between clinical and laboratory parameters and the need for validated biomarkers for monitoring disease activity. More studies and efforts are needed for better characterization and standardization of EGE diagnosis criteria, for personalized therapeutic options and patient access to the most advanced available therapies.

## Figures and Tables

**Table 1:** Abnormal laboratory tests at EGE diagnosis (2017).

Laboratory Test	Value	Reference Values
Total serum IgE	628 IU/mL	<100 IU/mL
Food specific IgE		<0.35 kU/L
cow milk	53 kU/L	
wheat	18 kU/L	
eggs white	13 kU/L	
egg yolk	7.5 kU/L	
turkey meat	23 kU/L	
chicken meat	12.8 kU/L	
pork meat	58 kU/L	
beaf meat	74 kU/L	
seafood	15.4 kU/L	
25-hydroxycholecalciferol	10.96 ng/mL	30–100 ng/ml
Total serum protein	5.19 g/dL	>6.6 g/dL
Iron	53.2 μg/dL	59–158 μg/dL
Ferritin	20.1 μg/L	30–400 μg/L
Total serum calcium	8.14 mg/dL	8.6–10.2 mg/dL
Eosinophil cationic protein (ECP)	>200 μg/L	<13.3 μg/L

**Table 2:** Summary report of increased serum specific IgE reproduced from Allergy Explorer Test (2019) (ELISA multiplex method, Macro array Diagnostics).

Food-Specific IgE		
Name	Allergen Component	Value (N < 0.3 kU_A_/L)
Cattle meat	Bos d 6	6.44 kU_A_/L
Wheat	Tri a	6.23 kU_A_/L
Egg white	Gal d_withe	4.87 kU_A_/L
Cultivated rye	Sec c_flour	4.93 kU_A_/L
Egg white	Gal d 3	2.52 kU_A_/L
Oat	Ave s	2.34 kU_A_/L
Sheep meat	Ovi a_meat	2.18 kU_A_/L
Peanut	Ara h 9	1.20 kU_A_/L
Kiwi	Act d 2	1.37 kU_A_/L
Melon	Cuc m	1.75 kU_A_/L
Cow milk	Bos d_milk	0.52 kU_A_/L
	Respiratory-Specific IgE	Value (N < 0.3 kU_A_/L)
Pig	Sus d_epithelia	3.76 kU_A_/L
Cat	Fel d 2	3.07 kU_A_/L
Guinea pig	Cav p	2.14 kU_A_/L
Silver birch	Bet v 6	2 kU_A_/L
Hamster	Cir c	1.79 kU_A_/L
Dog	Can f 3	1.69 kU_A_/L
Oak	Que r	1.59 kU_A_/L
Goat epithel	Cap h_epithelia	1.54 kU_A_/L
Cattle epithel	Bos d_epithelia	1.36 kU_A_/L
Rat	Rat n	1.25 kU_A_/L
Cypress	Cup a 1	1.22 kU_A_/L

## Data Availability

Data are available at Allergology and Immunology Department, Colentina Clinical Hospital, Bucharest, Romania.
